# *In Vitro* Identification of Histatin 5 Salivary Complexes

**DOI:** 10.1371/journal.pone.0142517

**Published:** 2015-11-06

**Authors:** Eduardo B. Moffa, Maria A. A. M. Machado, Maria C. M. Mussi, Yizhi Xiao, Saulo S. Garrido, Eunice T. Giampaolo, Walter L. Siqueira

**Affiliations:** 1 The University of Western Ontario, Schulich School of Medicine & Dentistry, Department of Dentistry and Biochemistry, London, ON, Canada; 2 UNESP – Univ. Estadual Paulista – Araraquara Dental School, Department of Dental Materials and Prosthodontics, São Paulo, Brazil; 3 CEUMA University, Department of Prosthodontics, Sao Luis, MA, Brazil; 4 Department of Pediatric Dentistry, Orthodontics and Public Health, Bauru Dental School, USP – University of São Paulo, Bauru, SP, Brazil; 5 University of São Paulo, School of Dentistry, São Paulo, SP, Brazil; 6 UNESP – Univ. Estadual Paulista – Institute of Chemistry – Department of Biochemistry and Technological Chemistry, São Paulo, Brazil; Hans-Knoell-Institute (HKI), GERMANY

## Abstract

With recent progress in the analysis of the salivary proteome, the number of salivary proteins identified has increased dramatically. However, the physiological functions of many of the newly discovered proteins remain unclear. Closely related to the study of a protein’s function is the identification of its interaction partners. Although in saliva some proteins may act primarily as single monomeric units, a significant percentage of all salivary proteins, if not the majority, appear to act in complexes with partners to execute their diverse functions. Coimmunoprecipitation (Co-IP) and pull-down assays were used to identify the heterotypic complexes between histatin 5, a potent natural antifungal protein, and other salivary proteins in saliva. Classical protein–protein interaction methods in combination with high-throughput mass spectrometric techniques were carried out. Co-IP using protein G magnetic Sepharose TM beads suspension was able to capture salivary complexes formed between histatin 5 and its salivary protein partners. Pull-down assay was used to confirm histatin 5 protein partners. A total of 52 different proteins were identified to interact with histatin 5. The present study used proteomic approaches in conjunction with classical biochemical methods to investigate protein–protein interaction in human saliva. Our study demonstrated that when histatin 5 is complexed with salivary amylase, one of the 52 proteins identified as a histatin 5 partner, the antifungal activity of histatin 5 is reduced. We expected that our proteomic approach could serve as a basis for future studies on the mechanism and structural-characterization of those salivary protein interactions to understand their clinical significance.

## Introduction

Recent efforts in salivary research have resulted in the elucidation and characterization of the proteomes of the major gland human salivary secretions and whole saliva [1–6] by classical biochemical methods [7–10] as well as more advanced approaches [1,5,6,11–14]. Saliva contains a large array of proteins and peptides that have the potential to form complexes [15–19]. Biomolecular interactions play a critical role in the majority of cellular processes. Understanding the consequences of protein interactions is a crucial role for the development of novel therapeutics approaches [20]. The formation of complexes in biological systems involves ionic forces, hydrogen bonding, and/or hydrophobic interactions that can result in altered protein structure and can lead to new biological activities [21]. A previous study mapped protein interactions for 338 human bait proteins that were selected based on known disease and functional associations. Large-scale immunoprecipitation and mass spectrometry resulted in the identification of 6463 interactions between 2235 distinct proteins [20]. Other studies used the same approach to map protein-protein interactions in yeast, creating unique data sets for biology and extrapolation into mammalian biology [22,23]. Based on available data in regard to protein-protein interaction, another study used literature-mining algorithms to recover from Medline abstracts 6580 interactions among 3737 human proteins [24]. In saliva, salivary protein complexes also are described [15–17] and a suitable biological function for these complexes is to serve as a mechanism between salivary protein partners for protection from oral proteolysis; and/or to play a role in the delivery of salivary proteins to different locations in the oral cavity.

Based on these potential biological functions related to the salivary protein complexes, a comprehensive analysis of the salivary complexes present in saliva is needed to understand better the physiology of the oral cavity. Recently, histatin 1 was used as a target protein for the identification of salivary protein partners. In this study 43 proteins were identified as partners of histatin 1. In addition, it was found that these protein-protein interactions protect complex partners from oral proteolysis and modulates the biological activity of the proteins complexed [4]. Now, to continue these studies, histatin 5 was selected as our target protein. Histatin 5 was selected due to the abundance of this protein in saliva and its importance for maintaining oral homeostasis. In spite of histatin 1 and histatin 5 belonging to the same protein family, those proteins present significant differences in post-translational modification and biological activities. For example, histatin 1 is phosphorylated in the second amino acid residue while histatin 5 is un-phosphorylated. Histatin 5 is described as the major histatin related to the killing of an opportunistic fungus called *Candida albicans* [25], while histatin 1 represents the strongest histatin in relation to inhibition of enamel demineralization [26]. Based on this difference between those two abundant salivary proteins, the aim of this study was to identify the heterotypic complexes between histatin 5 and other salivary proteins in saliva by using classical protein-protein interaction methods in combination with mass spectrometric analysis. Further, this study aimed to evaluate the biological function of histatin 5 and some of its partners when complexed. As a final outcome, the present study intends to develop a high-throughput platform combining classical protein-protein interaction methods and mass spectrometry for rapid identification of novel protein interactions with a “bait” protein (e.g. histatin 5) of interest.

## Material and Methods

### Ethics approval and human participants

This study was approved by the Research Human Ethics Board of The University of Western Ontario (review number 16181E); moreover, all the volunteers signed consent forms. Saliva samples were collected from four healthy, nonsmoking adult volunteers, ranging in age from 22 to 33 years old (two males and two females). These individuals did not exhibit signs of gingivitis, periodontal disease, active dental caries, or any other oral or systemic condition that could affect saliva composition.

### Parotid collection

To minimize circadian effects, saliva samples were collected between 9:00 and 11:00 A.M. A total volume of 5 mL of saliva was collected [27]. Parotid saliva was stimulated with sugar-free sour candy and collected with a Carlson–Crittenden device and kept on ice during the collection procedure. A pool of parotid saliva from the four subjects was used in all experiments. The total protein concentration was measured by the bicinchoninic acid assay (Pierce Chemical, Rockford, IL, USA) using BSA as a protein standard.

### Coimmunoprecipitation assay using magnetic beads

Fifty microliters of PureProteome^™^ protein G magnetic beads suspension (Millipore, Billerica, MA, USA) was mixed with 500 μL of binding buffer (PBS, pH 7.5) for 5 min. Binding buffer was removed using the magnetic MagRack6^™^ leaving only the magnetic beads in the polypropylene microcentrifuge tube. Next, 20 μL of mouse anti-histatin 3/5 monoclonal antibody (catalogue number: CABT-15413MH, Santa Cruz Biotechnology, Inc., Santa Cruz, CA, USA), which presents specificity for common region of histatin 3 and histatin 5 [28,29], in 200 μL of binding buffer was added onto the beads and incubated at 4°C for 60 min, allowing binding of the antibodies to the beads. After incubation, the beads were washed three times with 500 μL of binding buffer to remove unbound antibodies. To identify the histatin 5 complexes with salivary proteins, the beads were previous incubated at 4°C for 24 h with 100 μg of histatin 5 (SynPeptide Co., Ltd, Shanghai, China) in 400 μL of binding buffer which assure only histatin 5 present in the reaction antigen-antibody. After incubation the beads were washed wit h PBS. Finally, histatin 5- coated beads were incubated with 300 μg of dried parotid saliva resuspended in 400 μL of binding buffer at 4°C for 24 h. After incubation, the supernatant containing the nonbound fraction was removed and kept on ice for further analysis. Nonspecific proteins were washed with 500 μL of wash buffer (PBS, pH 7.5). This procedure was repeated three times. Each time, the supernatant was collected and kept on ice. Coimmunoprecipitated proteins were eluted with 50 μL of elution buffer (2.5% acetic acid) added onto the beads and incubated at 4°C for 15 min. This procedure was also repeated three times. The protein concentration of elution fractions was subsequently determined using the bicinchoninic acid protein concentration assay. Negative control experiments were carried out in the same manner, replacing antibodies with 200 μL of binding buffer. Positive control experiments were carried out in the same manner except 300 μg of parotid saliva protein was replaced with 100 μg of histatin 5.

### Pull-down assay

For pull-down experiments, immunopure immobilized streptavidin beads in 50 μL of 50% agarose slurry (Pierce Biotechnology–Thermo Scientific, Rockford, IL, USA) was incubated with 50 μg biotinylated histatin 5 (200 μL) for 2 h on ice with gentle agitation followed by centrifugation at 1250 × g for 1 min. Next, 300 μg of lyophilized parotid saliva dissolved in pH 7.0 binding buffer was added to the beads and incubated for 4 h at 4°C, with gentle agitation. Beads containing histatin 5 protein complexes were centrifuged at 1250 × g for 1 min and washed four times with 250 μL phosphate-buffered saline containing 75 mM NaCl, pH 5.0. This was followed by adding 200 μL of elution buffer (phosphate-buffered saline containing 75 mM NaCl, pH 2.8) incubated for 10 min and centrifuged at 1250 × g for 1 min. This last step was repeated five times to release histatin 5 protein complex. The 1 mL eluate sample was dried in a rotary evaporator (Eppendorf, Waltham, MA, USA), tryptic digested and subjected to MS analysis. Control experiments were carried out in the same manner excluding biotinylated histatin 5 or 300 μg of parotid saliva.

### SDS-PAGE and In-Gel protein digestion

A total of 10 μg of histatin 5 complex samples was dissolved with 20 μL of sample buffer (0.125 M Tris−HCl, 4% SDS, 2% glycerol, 10% 2-mercaptoethanol). After boiling for 5 min, the samples were placed directly in one of the 18 wells of a precast 12% SDS-PAGE lane (Bio-Rad, Hercules, CA). Gel electrophoresis was carried out to optimize the separation of individual proteins. During electrophoresis, the voltage was kept constant at 80 V. As a control 10 μg of histatin 5 and parotid saliva were used and 5 μL of high and lower MW standard (Precision Plus protein standard, Bio-Rad, Hercules, CA) were directly loaded on the gel. After the gel staining procedure (Pierce^®^ Color Silver Stain Kit), gel bands were excised with a razor blade and subsequently cut in three parts. Excised gel slices were each cut into three approximately 1 mm pieces. Gel pieces were then subjected to a modified in-gel trypsin digestion procedure [30]. The trypsinization was carried out in 25 mM ammonium bicarbonate solution containing 12.5 ng/μL of modified sequencing-grade trypsin (Promega, Madison, WI). Peptide extraction was achieved by multiples wash and hydration steps.

### In-solution digestion

Eluate samples from both experimental methods were dried in a rotary evaporator, denatured and reduced for 2 h by the addition of 200 μL of 4 M urea, 10 mM DTT, and 50 mM NH_4_HCO_3_, pH 7.8. After four-fold dilution with 50 mM NH_4_HCO_3_, pH 7.8, tryptic digestion was carried out for 16 h at 37°C, after the addition of 2% w/w sequencing-grade trypsin (Promega, Madison, WI, USA).

### MS analyses

Mass spectrometric analyses were carried out with a LTQ-Velos (Thermo Scientific, San Jose, CA, USA), which allows in-line LC with the capillary-fused silica C18 column (column length 10 mm, column id 75 μm, 3-μm spherical beads, and 100 Å pore size) linked to a mass spectrometer using an ESI in a survey scan in the range of m/z values 390–2000 MS/MS. All samples either obtained by coimmunoprecipitation or pull-down assay were dried by rotary evaporator and resuspended in 15 μL of 97.5% H_2_O/2.4% ACN/0.1% formic acid and then subjected to RP LC-ESI-MS/MS. The nanoflow RP-HPLC was developed with a linear 65-min gradient ranging from 5 to 55% of solvent B (97.5% ACN, 0.1% formic acid) at a flow rate of 300 nL/min with a maximum pressure of 280 bar. Electrospray voltage and the temperature of the ion-transfer capillary were 1.8 kV and 250°C, respectively. Each survey scan (MS) was followed by automated sequential selection of seven peptides for CID, with dynamic exclusion of the previously selected ions. The obtained MS/MS spectra were searched against human protein databases (Swiss Prot and TrEMBL, Swiss Institute of Bioinformatics, Geneva, Switzerland, http://ca.expasy.org/sprot/) using SEQUEST algorithm in Proteome Discoverer 1.3 software (Thermo Scientific). Tryptic and nontryptic peptides were considered. An additional inclusion criterion for positive identification and characterization of proteins was that the same constituent was found inboth methods, coimmunoprecipitation and pull-down assay.

### Histatin 5 heterotypic complex reconstitution

A total of 40 nmol/mL of synthetic histatin 5, was added to 40 nmol/mL of salivary amylase (MP Biomedicals, Solon, OH, USA) prepared in 50 mM NaCl, pH 7.0 to mimic the ionic strength of saliva, and incubated at 37°C for 2 h with gentle agitation. *In vitro* histatin 5/amylase salivary complex was recovered using a disposable PD-10 desalting Sephadex column (GE Healthcare). After recovery, the salivary complex was electrophoresed under 12% native PAGE conditions to confirm histatin 5/amylase salivary complex formation.

### Hydrolysis of starch by in vitro histatin 5/amylase complex

Amylase activity was determined as described previously [4]. An equivalent of 40 nmol/mL histatin 5/amylase complex was incubated with 1% starch solution in 20 mM phosphate buffer, pH 7.0, for 5 min at 30°C. The reaction was interrupted by the addition of an alkaline solution of 1% dinitrosalicylic acid, and the mixture was maintained in boiling water for 5 min. The mixture was diluted with distilled water and the absorbance at 530 nm was determined with a microplate reader (Bio-Rad, Inc., USA). Histatin 5 and amylase were also assessed separately. All experiments were carried out in triplicate.

### Fungal-killing assay of in vitro histatin 5/amylase complex

*Candida albicans* (ATCC 10231) colonies were picked from a Sabouraud Dextrose Agar (SDA; Difco Laboratories, Detroit, MI, USA) plate (<1 week old) and suspended in 5 mM potassium phosphate buffer, pH 7.0, to a final OD 620 nm of approximately 0.55. From this suspension, 50 μL was added to 50 μL of a serial dilution series of histatin 5, histatin 5/amylase complex (see histatin 5 heterotypic complex reconstitution session for details), and amylase in a 96-well polypropylene microtiter plate followed by serial dilution and incubation again with *Candida albicans*. The initial concentration during the dilution series of isolated histatin 5 or complexed with amylase was 66 μmol/mL. In addition, to mimic the *in vivo* complex formation, a second histatin 5/amylase complex was formed in direct contact with *C*. *albicans*, using an initial concentration of 66 μmol/mL. This experiment mimics the formation of the salivary complex in the presence of *C*. *albicans*. All wells were incubated for 1.5 h at 37°C. Afterwards, 50 μL from selected wells was diluted in 9 mL PBS, pH 7.0 and a 25 μL aliquot of the diluted suspension was plated on SDA. After 48 h of incubation at 30°C, cell viability was assessed by colony counting, using comparisons with the number of cells in a control sample incubated without the presence of salivary proteins, complexed or not [31]. This experiment was carried out in triplicate.

## Results

### Histatin 5 protein partner’s identification

Our first goal was to identify the salivary proteins that complex with histatin 5 and two protein-protein interaction methods were used. In addition, in-gel and in-solution trypsinization followed by mass spectrometry protein identification were carried out. The results obtained by in-gel and in-solution trypsinization are shown in Table 1.

**Table 1:** In-Solution and In-Gel salivary protein identification when complexed with histatin 5.

Accession	Description	In-Solution	In-Gel	calc. pI	M.W. (KDa)
Q9HBU2	Lim-homeobox transcription factor LHX3	X		8.94	43.6
Q8TAX7	Mucin-7	X	X	8.78	39.1
Q8N355	IGL@ protein	X		6.37	24.8
Q8IXZ2	Zinc finger CCCH domain-containing protein 3	X		10.95	101.9
Q6ZUJ8	Phosphoinositide 3-kinase adapter protein 1	X		5.40	90.3
Q6ZSZ6	Teashirt homolog 1	X		7.06	117.8
Q53F69	MUF1 protein variant (Fragment)	X		8.22	66.5
Q19KS2	Lactoferrin (Fragment)	X	X	9.03	39.1
P61626	Lysozyme C	X	X	9.16	16.5
P23280	Carbonic anhydrase 6	X		7.02	35.3
P20930	Filaggrin	X		9.25	434.9
P15516	Histatin-3	X		10.10	6.1
P15515	Histatin-1	X		9.13	7.0
P12273	Prolactin-inducible protein	X		8.05	16.6
P08493	Matrix Gla protein	X	X	9.67	12.3
P04745	Alpha-amylase 1	X	X	6.93	57.7
P02812	Basic salivary proline-rich protein 2 (Fragment)	X		12.18	37.3
P01876	Ig alpha-1 chain C region	X	X	6.51	37.6
P00451	Coagulation factor VIII	X		7.36	266.8
H7BYZ0	Mucin-5B	X		6.80	574.3
H0Y8I1	Probable cation-transporting ATPase 13A2 (Fragment)	X		8.60	28.1
H0Y818	DNA mismatch repair protein Mlh1 (Fragment)	X		6.13	75.8
F8WBZ4	Unconventional myosin-VIIb	X		8.34	107.7
D3DSS6	Dedicator of cytokinesis 5, isoform CRA_a	X		7.90	213.9
C6KXN3	Lambda light chain of human immunoglobulin surface antigen-related protein (Fragment)	X	X	5.54	24.7
C3PTT6	Pancreatic adenocarcinoma upregulated factor	X	X	7.39	21.6
C0JYZ2	Titin	X		6.52	3711.4
B6DP75	Estrogen receptor alpha splice variant (Fragment)	X		9.85	4.8
B5MDS5	Adenomatous polyposis coli protein 2	X		9.38	213.7
B4DVE1	Galectin-3-binding protein	X		5.47	64.1
B1AME9	Ubiquitin-conjugating enzyme E2, J2 (UBC6 homolog, yeast) (Fragment)	X		9.70	32.5
P02808	Statherin	X		8.47	7.3
B7Z4X2	Lactoferroxin-C		X	7.85	39.1
Q9NPP6	Immunoglobulin heavy chain variant (Fragment)		X	6.13	44.8
Q4G0R1	PIBF1 protein		X	5.53	83.1
P22079	Lactoperoxidase		X	8.62	80.2
Q5DT20	Hornerin		X	10.02	282.2
P08123	Collagen alpha-2(I) chain		X	8.95	129.2
D6W625	Chromatin assembly factor 1, subunit A (P150), isoform CRA_a		X	5.94	106.9
Q4KWH8	1-phosphatidylinositol 4,5-bisphosphate phosphodiesterase eta-1		X	7.74	189.1
H0YG08	Dedicator of cytokinesis protein 4		X	7.91	127.3
Q59EE7	Pro-alpha-1 type V collagen variant (Fragment)		X	5.00	178.4
P02788	Lactotransferrin		X	8.12	78.1
Q8N5K4	IGHA1 protein		X	6.68	53.3
P01877	Ig alpha-2 chain C region		X	6.10	36.5
Q8N5F4	IGL@ protein		X	5.34	24.9
Q8TDL5	Long palate, lung and nasal epithelium carcinoma-associated protein 1		X	7.23	52.4
F8VV32	Lysozyme C		X	9.07	16.5
F5GWP8	Junction plakoglobin		X	5.19	66.3
Q9UGM3	Deleted in malignant brain tumors 1 protein	X	X	5.44	260.7
Q96DR5	BPI fold-containing family A member 2		X	5.59	27.0
H0YMM1	Annexin (Fragment)		X	5.91	16.4

Note. Proteins described listed were identified in both, Co-IP and Pull-Down assay methods.

**Fig 1:**
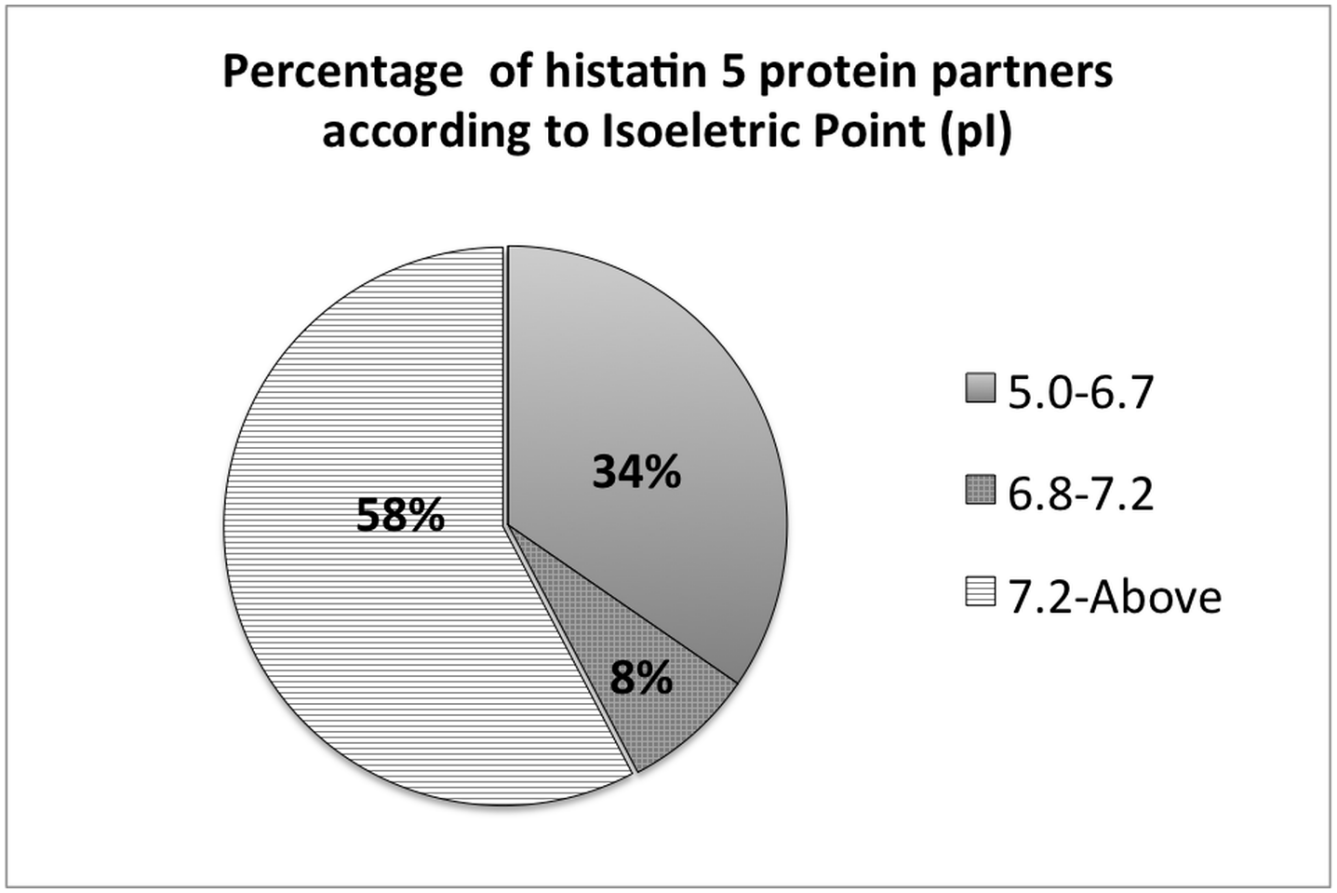
Percentage of histatin 5 protein partners according to Isoelectric Point (pI). The histatin 5 protein partners were divided according to the pI ranging from 5.0 to 6.7, 6.8 to 7.2 or above 7.2.

The LC-ESI-MS/MS analysis of those potential histatin 5 protein partners obtained by both Co-IP and pull-down techniques resulted in the identification of 83 different proteins that came from the “in-solution” and “in-gel” experiments. However, from the total of 83 proteins only 52 were present in both methods and were considered real partners from histatin 5. Moreover, those 52 different proteins displayed molecular weights ranging from 6.1 kDa to 3711.4 kDa (Table 1). The identified proteins were also grouped according to their isoelectric points (Fig 1). Clearly, more than 50% of all these proteins have isoelectric points above 7.2 and therefore exhibit a positive charge at a pH ranging between 6.8 to 7.2, the pH conditions that largely prevail in the oral cavity. Only one-third of the identified histatin 5 protein partners exhibited acidic characteristics ranging in pI between 5.0 to 6.7 (Fig 1).

### Biological activities of in vitro histatin 5/amylase complex

**Fig 2:**
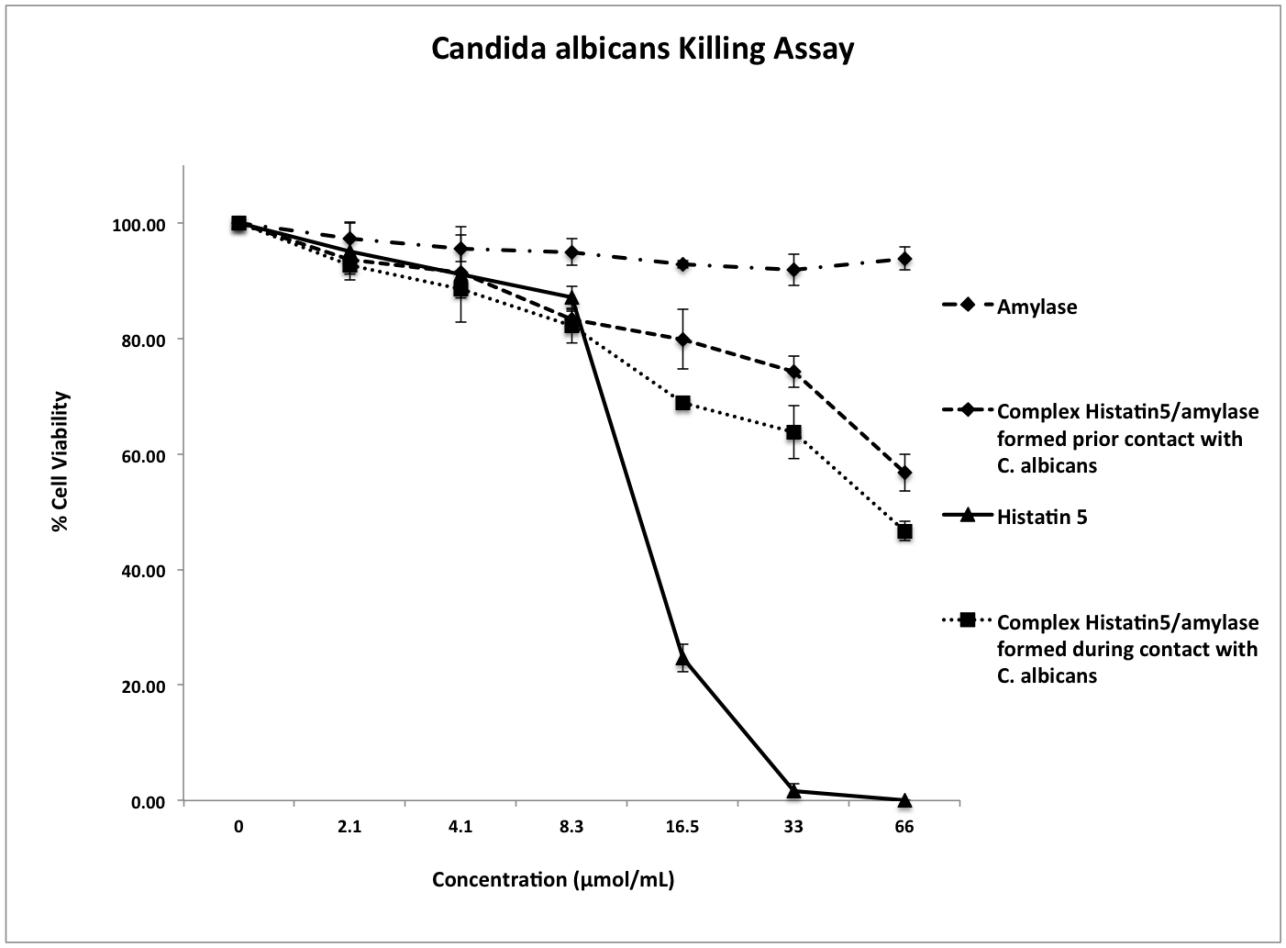
*C*. *albicans* killing Assay. *C*. *albicans* killing activity of histatin 5, amylase, histatin5/amylase complex prior incubate with *C*. *albicans* and histatin 5/amylase incubation with *C*. *albicans*. X-axis represents the concentration of histatin 5 complexed (or not). The complex was made based on 1:1 number of molecules from histatin 5 and amylase. Bars represent standard deviation of the mean, calculated from three independent experiments.

Among the 52 salivary proteins that complex with histatin 5, salivary amylase was selected to test the hypothesis that complexed proteins could have their biological role modified. First, histatin 5/amylase complex was subject to a *Candida albicans* killing assay, since histatin 5 is the most potent antifungal protein from the histatin family. Isolated histatin 5 and amylase were also tested in the same experiment. As expected, amylase alone, in the same tested concentrations as histatin 5/amylase complex and histatin 5 alone, showed no antifungal activity (Fig 2, p>0.05) while histatin 5 showed the higher antifungal activity in all tested concentrations (Fig 2, p<0.05). Moreover, the histatin 5/amylase complex formed in the presence of *C*. *albicans*, which tried to mimic the complex formation in the oral cavity, demonstrated killing activity similar to that of the histatin 5/amylase complex formed prior to incubation with *C*. *albicans* (Fig 2, p>0.05).

**Fig 3:**
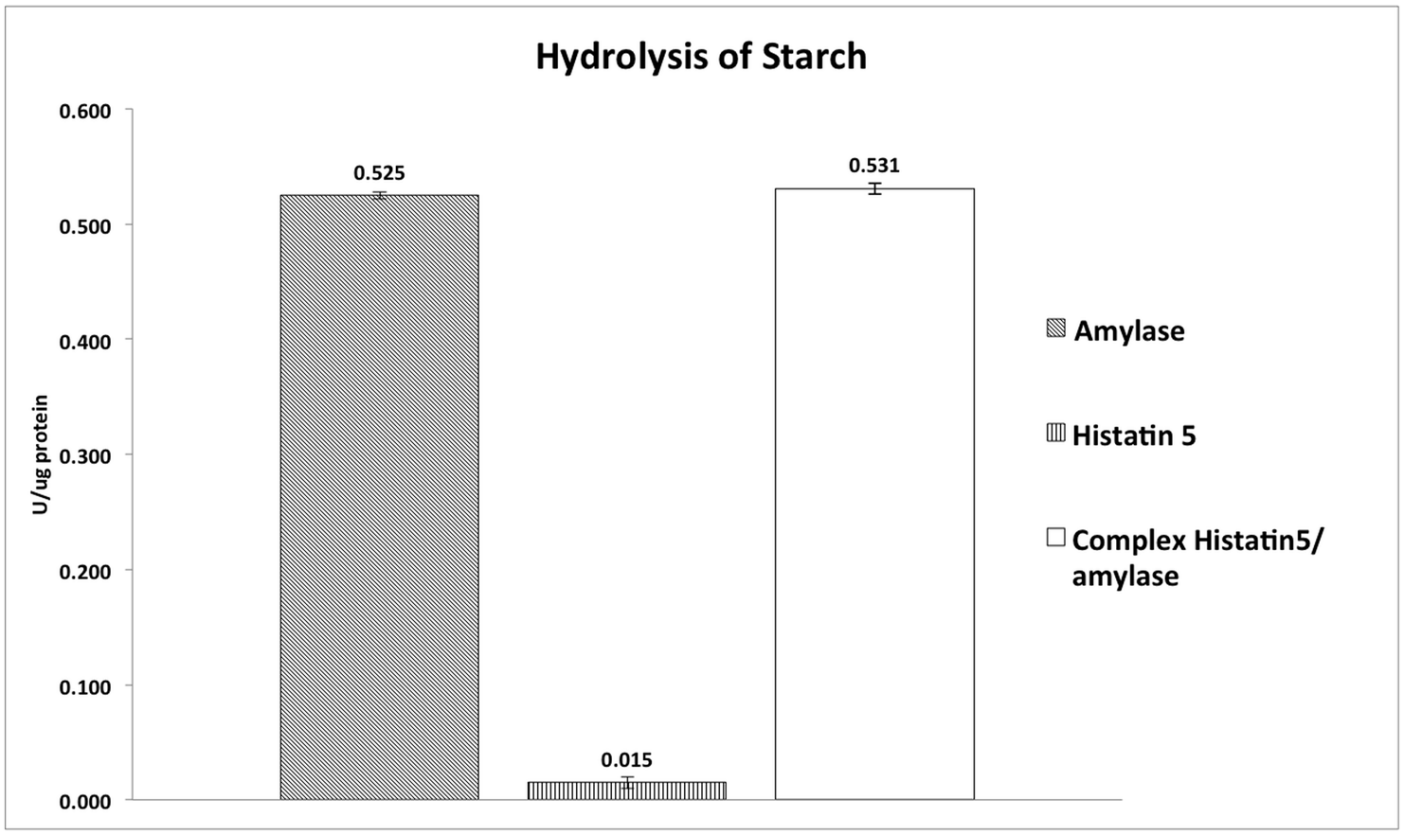
Hydrolysis of Starch. Hydrolysis of starch by histatin 5, amylase and histatin 5/amylase complex. Different lower case letters denote statistical difference according to Tukey’s test. Bars represent standard deviation of the mean, calculated from three independent experiments.

Since amylase was selected to test the hypothesis that complexed proteins could have their biological role modified, a second functional assay, hydrolysis of starch, related to the biochemical properties of amylase in saliva was also carried out. Amylase activity was measured when amylase was complexed or not with histatin 5. Our results showed no statistical difference between histatin 5/amylase complex and amylase alone (p>0.05). As expected, histatin 5 alone showed negligible starch hydrolysis activity (Fig 3, p<0.05).

## Discussion

The histatins are transcription products of two gene *loci* on chromosome 4 [32]. Histatin 1 is generated from HIS 1 gene while histatin 5 is a degradation product from histatin 3 that is generated from the HIS2 gene [33]. It should be noted that histatin 1, a neutral molecule, is a phosphorylated protein with 38 amino acids while histatin 5 is not phosphorylated and has only 24 amino acids. It seems that histatin 1 has affinity for anion exchangers due to the strong negative charge contributed by the serine phosphate at residue 2; in contrast histatin 5 has a net positive charge of 5, is basic in nature, and is not retained on anion exchangers [32]. In addition, several short and linear proteins do not have a folded structure in solution but after binding to a specific receptor on host cells, they may assume a secondary structure [34]. The structural flexibility of histatins may permit them to maintain an unordered state in the aqueous environment of the oral cavity, but adopt a helical conformation in a hydrophobic environment. Histatin 5 in a non-aqueous solvent assumes a largely alpha-helix conformation, but when in water or phosphate buffer (pH 7.4) the predominant form is a random coil shape [35]. The postulated *in vivo* functions for histatins include modulation of mineral formation (Histatin 1), as well as potent antifungal activities, where histatin 5 is the strongest one following by histatin 3 and 1, respectively [13,36]. In addition, our approach to study of a complex is based on future drug delivery for oral therapeutics, where histatin 5, a degradation product of histatin 3 and a small protein, could easily be incorporated into a drug delivery system, when compared with more structured proteins such as histatin 1 (phosphorylated and with 38 aminoacids). It is also known that even after early degradation, fragments of histatin 5 exhibit considerable antifungal activity [37,38].

Other groups have studied potential salivary protein–protein interaction [15–19]. For example, they have shown interaction of mucin 5B (MG1) or mucin 7 (MG2), high- and low-molecular weight salivary mucins, respectively, with such classical salivary proteins as amylase, acidic PRP 2, basic PRP 3, statherin, SIgA, lactoferrin, and histatin 1 by using a yeast two-hybrid system or far-Western blots [4,15–17,19].

Histatin 1 interactome has been recently explored, demonstrating that it is able to interact with 43 different proteins that influence different biological functions such as immune response, antimicrobial and metabolic processes, biomineralization and others. Among the 43 identified proteins, just 6 had been reported in previous studies using classical protein-protein interaction methods [7,39,40] leading to a discovery of 37 new proteins that interact with histatin 1 [4]. In our new exploration, it was possible to identify that histatin 5 may complex 52 different proteins present in saliva (Table 1). It is important to highlight that the total number of proteins came from the in-solution and in-gel digestion, improving the sensitivity and providing a more comprehensive identification, since gel digestion alone will not provide all the proteins/peptides from the gel bands [30]. In addition, co-immunoprecipitation was used as a primary method followed by mass spectrometry to identify putative histatin 5 complexes. Since it is well recognized that false positives can occur in any protein-protein interaction methods, pull-down assay was used as a second and validated method to identify proteins that complex histatin 5. The 52 proteins listed in Table 1 were identified with these two different methods. When a detailed analysis of the potential biological function of these proteins in the oral cavity was made based on information from protein annotation databases (e.g. Swissport; Uniprot), more than 50% of the identified proteins as partners of histatin 5 are proteins related to the metabolism process. The major source of these metabolic proteins is the host cells from the oral mucosa. Also significant was the number of antimicrobial proteins (20%) as histatin 5 complex partners. These include lysozyme C, carbonic anhydrase 6, histatin 3 and lactoperoxidase. These findings open avenues for a potential synergism effect on antimicrobial properties in the oral cavity. However, studies are required to investigate it further. When we compared the salivary proteins that complex with histatin 1 [4] with the identified proteins that complex with histatin 5, a total of 14 proteins were common for both. To our surprise the common proteins binding to histatin 1 and 5 are not the abundant salivary proteins such as alpha-amylase, mucin 7, mucin 5B, lysozyme C, carbonic anhydrase 6, histatin 3, histatin 1, prolactin-inducible protein, basic salivary proline-rich protein, Ig alpha-1 chain C region, statherin, lactoperoxidase, lysozyme C, deleted in malignant brain tumors 1 protein (G340) [41]. It is important to highlight that even using a Carlson–Crittenden device to avoid the parotid saliva contamination; The presence of mucin in our experiments could be related to a contamination from oral mucosa. Previous studies have reported that MUC7 and MUC5B are strongly retained on buccal cells thereby showing its crucial role in pellicle formation on soft oral tissues [42–44]. Protein-protein interaction is a common fact that occurs both within tissues and cells and in some body fluids, such as saliva [18,19]. In our study, despite the identification of these new proteins that complex with histatin 5, a limitation of the study is identification of the site for the complex formation (i.e. inside the salivary glands or in the oral cavity). Nevertheless, we can speculate that these salivary complexes are formed inside the salivary glands and also in the oral cavity. Our hypothesis here is based on the dynamic process of saliva output. In our experimental approaches, both pull-down and co-IP, the complexes were formed when the bait protein (histatin 5) was first linked to the antibody (co-IP experiment) or to the streptavidin-biotin (pull-down). Thus, all potential complexes formed inside the parotid gland were not analysed in this study.

A major unresolved question in oral biology is the discrepancy between the presence of salivary proteins in glandular secretions (salivary gland) and the absence of most of these proteins in their intact form from freshly collected whole saliva [45]. For example, histatins form a major portion of the glandular proteins but they disappear rapidly as soon as they are mixed with whole saliva [45]. Consequently, some of the biological properties linked to these proteins also disappear [45]. Logically, proteolysis has been the major focus of investigations aimed at explaining these differences [45]. On the other hand, although histatins are highly susceptible to proteolysis, we have observed that histatin 1 remains intact in the whole saliva environment when combined with other salivary proteins, and retains its partial antimicrobial activity [4]. In addition, we have shown that binding to tooth enamel (hydroxyapatite) confers intact histatin 1 with resistance to proteolytic degradation, increasing their lifespan in the oral cavity [46]. The discovery of these two physiological protective approaches for susceptible salivary proteins can guide the development of external mechanisms that can achieve delivery to the target tissue (tooth enamel and/or oral biofilm) with significant increase of the lifetime of these proteins. Thus, the investigation of biological functions of these complexes is extremely relevant to the area of biotechnology applied to the oral cavity. The interaction between proteins can modulate or alter (increase or diminish) their functions in saliva. Herein, we have observed that when histatin 5 is complexed with amylase (prior to or in the presence of C. albicans) its antifungal effect is significantly diminished when compared with histatin 5 alone. These data suggest a real antimicrobial effect for histatin 5 in the oral cavity, meaning that histatin 5 is an important antifungal salivary protein but due to the complex formation with other salivary proteins, there is a diminished antifungal effect. In spite of this limited antifungal effect, for therapeutic applications and/or a drug delivery system, it is common to use concentrations well above the physiological range (50–100 fold higher) to reach the same biological activity as the protein alone. On the other hand, amylase enzyme activity related to hydrolysis of starch was not significant different when amylase was complexed with histatin 5. Again, we can speculate that these observations could be related to the molecular structure of the salivary proteins tested where amylase (60 kD) is significantly larger compared with histatin 5 (3 kD). Atomic scale molecular dynamics simulations can be used to explore the binding amino acid chain position between histatin 5 and amylase which in vitro promotes a modulation of the antifungal activity of histatin 5 (Fig 2) but did not alter amylase enzyme activity (Fig 3).

In summary, despite the limitations of our in vitro experiment, the described approach mimics the real situation for the salivary proteins in the oral cavity. Our results provide a basis for future studies on the mechanism and functional significance of those salivary protein interactions which can lead to the development of an external protein delivery system; one that can reach the target tissue (tooth enamel and/or oral biofilm) with significant increase of the protein resistance to proteolytic degradation and maintenance of biological function.
